# Autophagy Regulates VDAC3 Ubiquitination by FBXW7 to Promote Erastin-Induced Ferroptosis in Acute Lymphoblastic Leukemia

**DOI:** 10.3389/fcell.2021.740884

**Published:** 2021-11-15

**Authors:** Ting Zhu, Bo Liu, Di Wu, Gang Xu, Yang Fan

**Affiliations:** ^1^ Pediatric Department, Shengjing Hospital of China Medical University, Shenyang, China; ^2^ Medical Research Center, Shengjing Hospital of China Medical University, Shenyang, China; ^3^ Liaoning Key Laboratory of Research and Application of Animal Models for Environmental and Metabolic Diseases, Shengjing Hospital of China Medical University, Shenyang, China

**Keywords:** acute lymphoblastic leukemia (ALL), autophagy, ferroptosis, erastin, ubiquitination

## Abstract

**Background:** The discovery of ferroptosis is a major breakthrough in the development of cancer treatments. However, the mechanism by which ferroptosis contributes to acute lymphoblastic leukemia (ALL) is to be clarified. Here, we explored erastin-induced ferroptosis in ALL cells and the impact of autophagic activity on this process.

**Materials and Methods:** Cell viability was evaluated in various ALL cell lines following erastin treatment by the MTS assay, while cell death was evaluated via a trypan blue assay. Immunoblotting and quantitative real-time PCR were used to detect protein and mRNA expression, respectively. The UbiBrowser database was used to predict the E3 ligase of VDAC3, which was confirmed by immunoprecipitation. The role of FBXW7 in erastin-induced ferroptosis *in vitro* was evaluated via lentiviral-mediated silencing and overexpression. ALL xenograft mice were used to observe the impact of autophagy on erastin-induced ferroptosis.

**Results:** Resistance to erastin-induced ferroptosis was higher in Jurkat and CCRF-CEM cells than in Reh cells. The sensitivity could be modified by the autophagy activator rapamycin (Rapa) and the autophagy inhibitor chloroquine (CQ). Rapa sensitized ALL cells to erastin-induced ferroptosis. In ALL xenograft mice, the combination treatment of Rapa and erastin resulted in longer survival time than those observed with erastin or Rapa treatment alone. VDAC3 was regulated by autophagy post-transcriptionally, mainly via the ubiquitin-proteasome system (UPS). FBXW7 was verified as a specific E3 ligase of VDAC3. *FBXW7* knockdown attenuated VDAC3 degradation by suppressing its ubiquitination, thereby increasing the sensitivity of ALL cells to erastin.

**Conclusion:** Autophagy regulated erastin-induced ferroptosis via the FBXW7-VDAC3 axis. Rapa sensitized ALL cells to erastin-induced ferroptosis both *in vitro* and *in vivo*. Our findings provide potential therapeutic targets for ALL.

## Introduction

Acute lymphoblastic leukemia (ALL) is a malignant clonal disorder of lymphoblastic hematopoiesis with high heterogeneity (Malard and Mohty 2020). Survival rates of ALL have improved remarkably by intensive induction chemotherapy, with complete remission (CR) rates of up to 80% (Bhojwani and Pui 2013; Inaba et al., 2013). However, relapse occured in 25–35% of patients (Bhojwani and Pui 2013). The CR rates following chemotherapy are significantly reduced after relapse, the efficacy of hematopoietic stem cell transplantation is also unsatisfactory, with 3-year survival rates of less than 10% in children (Gaynon 2005). These epidemiological data highlight the urgent need for novel ALL treatments, aimed at reducing relapse rates and optimizing overall survival.

Since chemotherapeutic agents mainly exert anti-leukemic effects by inducing apoptosis, the increase of anti-apoptotic activity in leukemia cells is one of the most common mechanisms for relapse. Ferroptosis is an iron-dependent form of programmed cell death (PCD) caused by the abnormal accumulation of lipid peroxidation products. It differs from other types of PCD, such as apoptosis, necroptosis, and autophagic cell death (Xie et al., 2016). Emerging evidence has revealed that ferroptosis is essentially a nexus between metabolism, redox biology, and diseases, including cancer. Triggering ferroptosis is a promising strategy for cancer therapy, particularly for aggressive malignancies that respond poorly to traditional therapies, and might be an effective approach to relapsed ALL (Dixon et al., 2012; Friedmann Angeli et al., 2019; Hassannia et al., 2019). Ferroptosis interacts with autophagy, which is crucial in the maintenance of cellular homeostasis (Nencioni et al., 2013; Dikic and Elazar 2018). Autophagy plays dual roles in the treatment of hematological malignancies. The activation of autophagy induces anti-tumor immunity to promote leukemia cell death; on the other hand, it causes drug resistance via maintaining the homeostasis in leukemia cells to help their survival (Auberger and Puissant 2017). Autophagy facilitates ferroptosis by the selective degradation of anti-ferroptosis regulators (Liu et al., 2020; Zhou et al., 2020; Li et al., 2021). It is therefore critical to clarify the interaction between autophagy and ferroptosis for the development of ALL treatment strategies.

Erastin is a classical ferroptosis activator. It affects the metabolic reprogramming of cells by closing the voltage-dependent anion channel (VDAC) in the mitochondrial membrane to produce reactive oxygen species (ROS), thereby inducing ferroptosis (Maldonado and Lemasters 2012; Reina et al., 2016). Recent studies have indicated that the stability of VDACs is a determinant of erastin-induced ferroptosis. In melanoma cells, NEDD4 degraded VDAC2/3 via the ubiquitin-proteasome system (UPS) to inhibit the sensitivity to erastin (Yang et al., 2020). However, in ALL, the relationship between autophagy and erastin-induced ferroptosis is not fully understood. Furthermore, the mechanism underlying VDAC protein regulation by autophagy and ferroptosis activators needs to be clarified.

F-box and WD repeat domain containing 7 (FBXW7) is a member of the F-box protein family, which serves the function of substrate recognition of E3 ubiquitin ligase. Substrates of FBXW7 include many critical proteins, e.g., Notch, Jun, c-Myc, and cyclinE (Welcker and Clurman 2008; Kourtis et al., 2015; Yeh et al., 2018). Accordingly, FBXW7 plays a pivotal role in multiple types of cancers including colorectal cancer, gastric cancer, lung cancer, and ALL (King et al., 2013; Huang et al., 2018; Xiao et al., 2018; Li et al., 2019). Our present work discovered and verified FBXW7 as a specific E3 ligase for VDAC3. We evaluated the regulatory effects of autophagy on the expression of VDAC3 via FBXW7 and the sensitivity of ALL cells to erastin-induced ferroptosis. Our study might be valuable in clarifying the roles of autophagy, FBXW7, VDAC3, and ferroptosis in ALL, further providing a possible alternative treatment for ALL.

## Materials and Methods

### Cell Culture

ALL cell lines, including Jurkat, CCRF-CEM, CEM/C1, Nalm6, Sup-B15, THP-1, HL-60, and Reh cells, and the human embryonic kidney cell line 293T, were purchased from the National Collection of Authenticated Cell Cultures (Shanghai, China). CCRF-CEM, CEM/C1, Nalm6, and Reh cells were cultured in RPMI 1640 medium (Gibco, Carlsbad, CA) containing 10% fetal bovine serum (FBS, Corning, New Zealand Sourced), and 1% penicillin–streptomycin (Gibco). Jurkat cells were cultured with RPMI 1640 containing 10% FBS, 1% GlutaMAX (Gibco), and 1% penicillin–streptomycin. THP-1 cells were cultured in RPMI 1640 containing 10% FBS, 0.05 mmol/l β-Mercaptoethanol (Gibco), and 1% penicillin–streptomycin. HL-60 and Sup-B15 cells were cultured in IMDM (Gibco) containing 20% FBS and 1% penicillin–streptomycin. 293T cells were cultured with Dulbecco’s modified Eagle’s medium (DMEM) (Gibco) containing 10% FBS and 1% penicillin–streptomycin. All cells were maintained at 37°C with 5% CO_2_, and saturated humidity. Cells in the logarithmic growth phase were used for subsequent experiments. All cells were identified by short tandem repeat (STR) profiling and were *mycoplasma* negative.

### Antibodies and Chemicals

The following antibodies were used at the indicated dilution for immunoblotting (IB) and immunoprecipitation (IP) analyses: VDAC3 (1:800 for IB, 3 µg for IP; Proteintech, Wuhan, China), FBXW7 (1:800 for IB; 3 µg for IP, Proteintech), Ubiquitin (1:800 for IB, Proteintech), and β-Actin (1:8,000 for IB, Proteintech). Horseradish peroxidase (HRP) labeled secondary antibody conjugates were purchased from Molecular Probes (Abbkine, Beijing, China). Erastin (APExBIO, Beijing, China), ferrostatin-1 (Fer-1), rapamycin (Rapa), MK-2206, chloroquine (CQ), 3-MA, bafilomycin A1 (BafA1), MG132, and imidazole ketone erastin (IKE) were obtained from Selleck Chemicals (Houston, TX).

### Cell Viability and Cell Mortality Assays

Cell viability was detected by the MTS assay (Promega, Madison, WI). Briefly, cells were equally seeded into 96-well plates with various concentrations of erastin (0.01, 0.1, 1, 10, and 100 μmol/l). DMSO (Sigma-Aldrich, St. Louis, MO) was used as a negative control. Cell viability was measured using the MTS kit at 24 h. Absorbance was tested at 490 nm using a microplate analyzer (BioTek, Winooski, VT). Cell mortality was detected by a trypan blue assay (Beyotime Biotechnology, Shanghai, China). In brief, cells were stained with trypan blue and counted under an inverted microscope. The blue-stained cells were considered dead, and five random fields per insert were counted. Cell mortality was calculated according to the following formula: cell mortality (%) = number of dead cells/total number of cells × 100.

### RNA Isolation and Quantitative Real-Time PCR

Total RNA was extracted using RNAiso Plus Reagent (TaKaRa, Dalian, China) and dissolved in 10 μl of RNase-free water. The purity and concentration were determined using the NanoPhotometer 50 (Implen, Munich, Germany). Next, 1 μg of total RNA was used for reverse transcription to synthesize cDNA using the PrimeScript RT Reagent Kit (TaKaRa). For quantitative real-time polymerase chain reaction (qRT-PCR), the amplification system consisted of 2 μl of cDNA, 1 μl of forward and reverse primers, 10 μl SYBR Premix Ex TaqII (2×), 0.4 µl of ROX Reference DyeⅡ (50×), and ddH_2_O for a total volume of 20 μl. The amplification conditions were as follows: initial denaturation at 95°C for 30 s, followed by 40 cycles of 95°C for 5 s and 60°C for 30 s. Relative expression levels were quantified using the 2^−ΔΔ^Ct method. The primer sequences were as follows (synthesized by GenScript, Nanjing, China): *FBXW7*, 5′-GGC CAA AAT GAT TCC CAG CAA-3′ (Forward), 5′-CCC TAC ATT GCA GAT GAG GCT C-3′ (Reverse); *VDAC3*: 5′-TTG TAC CGA ACA CAG GAA AGA AG-3′ (Forward); β-*Actin*, 5′-CAC CAT TGG CAA TGAGCGGTT C-3′ (Forward), 5′-AGG TCT TTG CGGATGTCCACG T-3′ (Reverse).

### Immunoblotting

For the immunoblotting analysis, cells were washed with phosphate-buffered saline (PBS) and lysed in radioimmunoprecipitation assay (RIPA) lysis buffer (Solarbio, Beijing, China) containing 1% PMSF (Solarbio) and 1% protease inhibitor cocktail (Sigma-Aldrich, St. Louis, MO). The cells were lysed on ice for 30 min, vibrated and mixed for 10 s every 10 min, and then centrifuged at 14,000 rpm for 30 min at 4°C. The bicinchoninic acid (BCA) method was applied to detect the protein concentration in the supernatant. Next, 30 µg of total protein samples were separated by 10% SDS-PAGE and transferred to polyvinylidene difluoride membranes; the membranes were incubated with primary antibodies (anti-VDAC3, anti-FBXW7, or Ubiquitin antibody) overnight at 4°C. Subsequently, the membranes were blocked with 5% skim milk and then incubated with HRP-conjugated goat anti-rabbit or goat anti-mouse IgG (H+L) as secondary antibodies for 2 h at room temperature. Immunoreactive protein bands were visualized using the ECL Detection Kit (Thermo Fisher Scientific, Waltham, MA).

### Immunoprecipitation and Ubiquitylation Assays

Cells were harvested and lysed with IP lysis buffer containing 1% PMSF and a 1% protease inhibitor cocktail. Approximately 1 mg of protein extract was used for each sample. First, the cell extract was combined with 3 μg of IP antibody (anti-FBXW7/anti-VDAC3, normal IgG) per sample on a rotating wheel at 4°C overnight. Next, the mixture was co-precipitated with 25 µl of protein A/G beads (MCE, Shanghai, China) on a rotating wheel at 4°C for 1 h. The beads were washed four times with IP lysis buffer, followed by the addition of 20 μl of 2× loading buffer at 100°C for 5 min at 100°C. The supernatants in the 2× loading buffer were prepared for subsequent analyses.

For ubiquitylation assays, cells were treated with 10 μmol/l MG132 for 6 h before harvesting. Cell extracts were incubated with the VDAC3 antibody and protein A/G beads on a rotating wheel for 2 h at 4°C. Subsequently, the beads bound proteins were eluted by boiling them in loading buffer and subjected to immunoprecipitation.

### Lentiviral Transfection

The FBXW7 overexpression lentiviral vector (pRRLSIN-cPPT-SFFV-MCS-3FLAG-E2A-EGFP-SV40-puromycin) and FBXW7 RNAi lentiviral vectors (pRRLSIN-cPPT-U6-shRNA-SFFV-EGFP-SV40-puromycin) were purchased from GeneChem (Shanghai, China). 293T cells, Jurkat cells, and Reh cells were infected following the manufacturer’s protocol. The cells were subjected to puromycin selection for 1 week, and those surviving cells were used for subsequent experiments.

### Tumor Xenografts in NOD/SCID Mice

NOD/SCID mice were purchased from HFK Bioscience (Beijing, China). The mice (males, 6–7 weeks old, and 19–21 g weight) were bred under pathogen-free conditions. The mice were provided free access to food and water in a 12 h light/12 h dark cycle animal facility. CCRF-CEM cells (5 × 10^6^/200 μl) were injected into the tail veins of mice. Flow cytometry (FCM) of peripheral blood (PB) was performed to detect huCD45^+^mCD45^–^ cells every week. PB from the orbital venous plexus was sampled and flushed in PBS solution. CCRF-CEM cells from PB were labeled with PerCP-conjugated anti-huCD45 (BioLegend, San Diego, CA) and FITC-conjugated anti-mCD45 (BioLegend) to determine the fraction of human ALL cells (huCD45^+^mCD45^–^) by FCM. The mice were treated after the disease burden was sufficient (>1% huCD45^+^/mCD45^–^ blasts detected in PB) (Teachey et al., 2006; Maude et al., 2012). The mice were randomly divided into four groups (8 mice per cage) and treated with rapamycin (2 mg/kg), IKE (20 mg/kg), rapamycin (2 mg/kg) + IKE (20 mg/kg), and vehicle (2% DMSO + 30% PEG300 + 68% normal saline) once every other day by i.p. injection for 2–3 weeks. The survival time for each group was recorded. All animal experiments were reviewed and approved by the Shengjing Hospital of China Medical University Institutional Animal Care and Use Committee (No: 2019PS162K).

### Statistical Analysis

All statistical analyses were performed using SPSS 25.0 software (Chicago, IL) and GraphPad Prism 8.0 software (San Diego, CA). Data are expressed as means ± SD. Quantitative data were analyzed using Student’s *t*-test for comparisons between two groups and one-way analysis of variance (ANOVA) with Bonferroni correction for comparisons among multiple groups. The survival curve was drawn by the Kaplan–Meier method and evaluated with the log-rank test. All experiments were performed at least three times, and a *p*-value < 0.05 was considered statistically significant.

## Results

### Autophagy Activation Increased the Sensitivity of Erastin-Resistant ALL Cells

**FIGURE 1:**
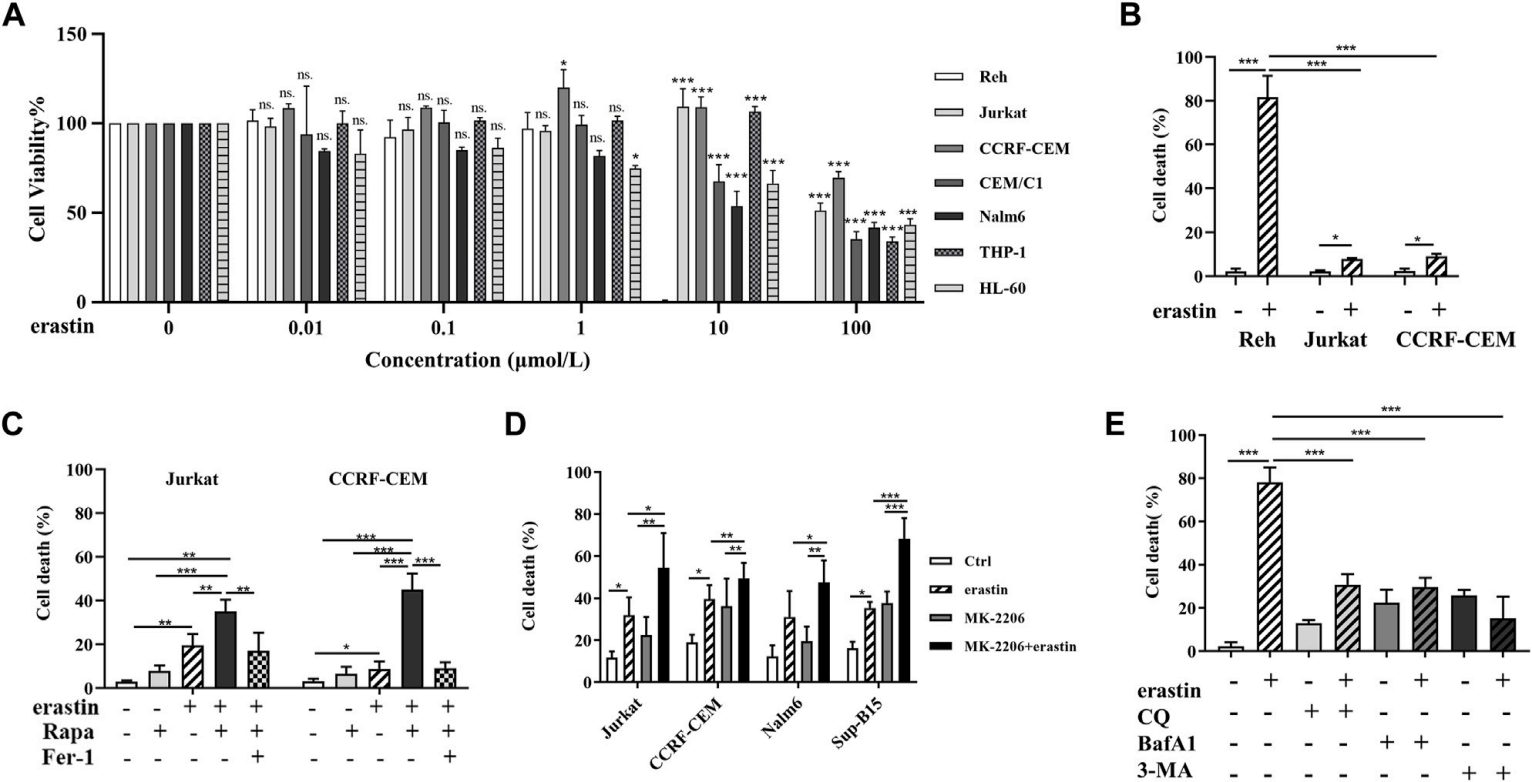
Erastin decreased cell viability and autophagic activity regulated erastin-induced ferroptosis in ALL cells. **(A)** ALL cells were treated with 0, 0.01, 0.1, 1, 10, and 100 μmol/l erastin for 24 h. The viability of ALL cells was measured via the MTS assay. **(B)** ALL cells were treated with erastin (20 μmol/l) for 24 h, trypan blue staining to evaluate cell death. **(C)** Jurkat cells and CCRF-CEM cells were treated with the indicated stimuli (200 nmol/l Rapa, 20 μmol/l erastin, 4 μmol/l fer-1, Rapa+erastin, and Rapa+erastin+fer-1) for 24 h and cell death was evaluated. **(D)** Jurkat, CCRF-CEM, Nalm6 and Sup-B15 cells were treated with the indicated stimuli (4 μmol/l MK-2206, 20 μmol/l erastin, and MK-2206 + erastin) for 24 h and cell death was evaluated. **(E)** Reh cells were treated with the indicated stimuli (20 μmol/l erastin, 20 μmol/l CQ, 4 μmol/l BafA1, 5 mmol/l 3-MA, CQ+erastin, BafA1+erastin, or 3-MA+erastin) for 24 h and cell death was evaluated. *p < 0.05, **p < 0.01, ***p < 0.001.

To determine the cytotoxic effect of erastin on ALL cell lines, we treated Reh, Jurkat, CCRF-CEM, CEM/E1, Nalm6, THP1, and HL-60 cells with various concentrations of erastin (0–100 μmol/l) for 24 h and assessed cell viability using MTS assays. As shown in Figures 1A,B, cell viability following erastin treatment varied among ALL cell lines. Reh cells exhibited the most sensitive response, while others displayed the resistance to erastin-induced ferroptosis.

To verify whether autophagy could regulate ferroptosis in ALL cells, the cell mortality of Jurkat, CCRF-CEM, and Reh cells were evaluated after treatment with erastin and Rapa or CQ. As shown in Figure 1C, the mortality rates of Jurkat and CCRF-CEM cells were significantly higher for the combination of Rapa with erastin than for Rapa or erastin alone (*p* < 0.001 for both). Jurkat and CCRF-CEM cells were further treated with the ferroptosis inhibitor ferrostatin-1 (Fer-1). Fer-1 attenuated the combined effect of Rapa and erastin in cell death, indicating that Rapa participated in the ferroptosis. In addition, Rapa promoted erastin-induced ferroptosis in other ALL cell lines (Nalm6 and Sup-B15 cells, in Supplementary Figure S1). These results indicated that Rapa promotes erastin-induced ferroptosis in ALL cells. Moreover, Jurkat, CCRF-CEM, Nalm6, and Sup-B15 cells were treated with another autophagy activator, MK-2206, and similar results were obtained (Figure 1D). These data indicated that autophagy activation could promote the erastin-induced ferroptosis in ALL cells. In contrast, autophagy inhibitors such as CQ, BafA1, and 3-MA reduced Reh cell mortality induced by erastin (*p* < 0.001 for all, Figure 1E). These findings demonstrated that activation of autophagy promoted the ferroptosis of ALL cells induced by erastin, and blockage of autophagy protected ALL cells from erastin-induced ferroptosis.

### Autophagy Activation Promoted Ferroptosis by Up-Regulating VDAC3 Expression

**FIGURE 2:**
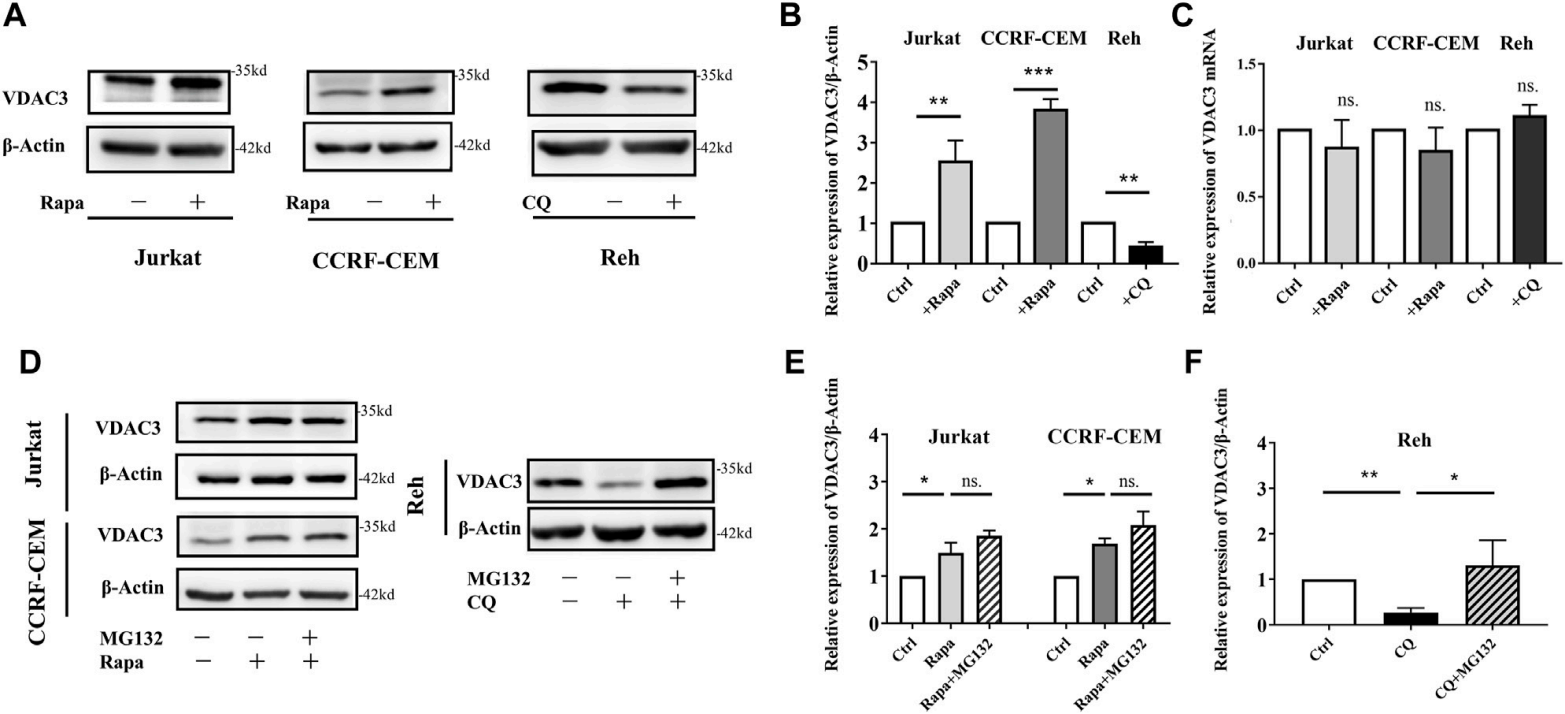
Autophagy activation promoted VDAC3 expression via proteosome degradation in ALL cells. **(A)** The expression levels of VDAC3 in Jurkat, CCRF-CEM, and Reh cells after Rapa and CQ treatment. The expression of β-actin was examined as a control. **(B)** Histogram of the relative expression levels of VDAC3 with different treatments. **(C)** Histogram of the relative mRNA expression levels of VDAC3 under different treatments. **(D)** Cells were treated in the absence or presence of 1 μmol/l MG132 and whole cell lysates were prepared for IB with a VDAC3 antibody. The expression of β-actin was examined as a control. **(E)** Histogram of the relative expression levels of VDAC3 in Jurkat and CCRF-CEM cells under different treatments. **(F)** Histogram of the relative expression levels of VDAC3 in Reh cells under different treatments. Results are means ± SD of three independent experiments. *p < 0.05, **p < 0.01, ***p < 0.001, ns. indicates no statistical significance.

VDAC3 is a direct target of erastin. The expression of VDAC3 was explored following Rapa administration in ALL cells. As shown in Figures 2A,B, in Jurkat and CCRF-CEM cells, VDAC3 expression increased substantially after Rapa treatment, and decreased significantly in Reh cells following CQ treatment (*p* < 0.05). VDAC3 expression in Reh cells was also higher after Rapa treatment while CQ treatment downregulated in Jurkat and CCRF-CEM cells (both *p* < 0.05, Supplementary Figure S2). Notably, despite substantial alterations in VDAC3 at the protein level, Rapa had no effect on mRNA expression (Figure 2C). The alteration of autophagic activity (by Rapa or CQ) did not affect the VDAC3 transcription, suggesting that autophagy contributed to the post-translational regulation of VDAC3. Protein degradation in eukaryotic cells occurs mainly via the autophagy lysosome pathway and UPS. Since the blockage of autophagy lysosome degradation did not preserve VDAC3 expression (Figure 2A), VDAC3 protein degradation was predicted mainly to be regulated by UPS. When cells were treated with autophagy regulators (CQ or Rapa) and MG132, as depicted in Figures 2D–F, MG132 inhibited the Rapa-induced VDAC3 expression in Jurkat and CCRF-CEM cells. VDAC3 expression in Reh cells increased significantly after combined treatment with CQ and MG132. These results supported the prediction that autophagy activation promotes the VDAC3 expression post-transcriptionally, mainly via the UPS. Next, we examined the specific mechanism underlying VDAC3 degradation via the UPS.

### Autophagy Activation Increased VDAC3 Expression via FBXW7

**FIGURE 3:**
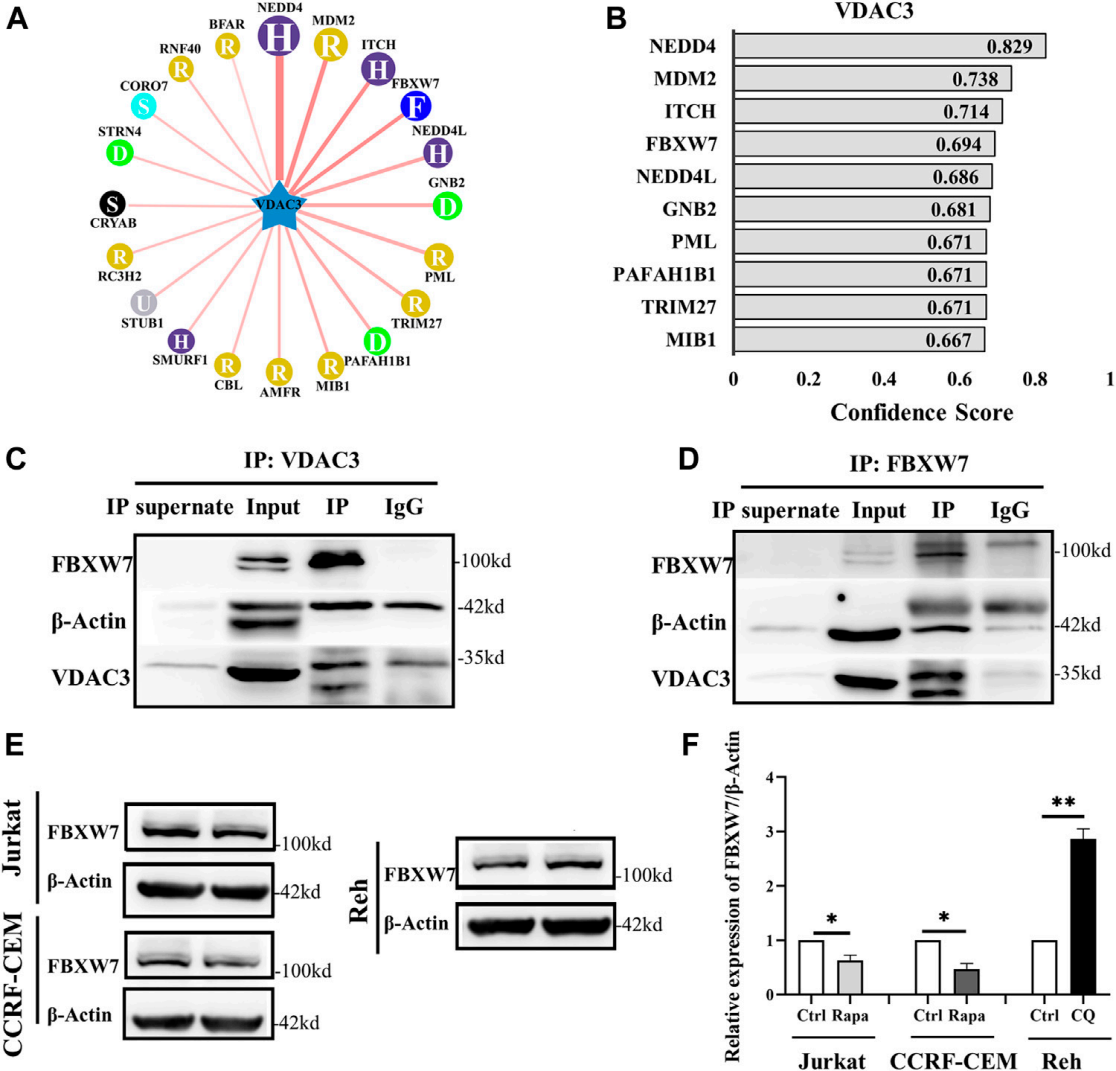
FBXW7 participated in the ubiquitination and degradation of VDAC3. **(A,B)** Top 10 E3 ligases of VDAC3. **(C)** Whole cell lysates were prepared and subjected to immunoprecipitation with either VDAC3 antibody or normal IgG antibody. A FBXW7 antibody was used for IB. **(D)** Whole cell lysates were prepared and subjected to immunoprecipitation with either the FBXW7 antibody or normal IgG antibody. A VDAC3 antibody was used for IB. **(E)** Comparison of expression levels of FBXW7 in Jurkat, CCRF-CEM, and Reh cells by IB. The expression of β-actin was examined as a control. **(F)** Histogram of the relative expression levels of FBXW7 under different treatments. Results are presented as means ± SD of three independent experiments. *p < 0.05, **p < 0.01.

Based on the results indicating that VDAC3 was regulated post-transcriptionally by autophagy via the UPS, the UbiBrowser database was used to predicted the E3 ligase of VDAC3 (Li et al., 2017). The top 10 E3 ligases were shown in Figures 3A,B. NEDD4 was reported to degrade VDAC2/3 via the UPS to inhibit the sensitivity of melanoma cells to erastin (Yang et al., 2020). We failed to show the interaction between ITCH/MDM2 and VDAC3 by co-immunoprecipitation (Co-IP) and IB assays. We then investigated whether FBXW7 could interact with VDAC3. As shown in Figures 3C,D, FBXW7 was enriched when VDAC3 was targeted by IP, while VDAC3 was enriched when FBXW7 was the target protein. This confirmed the interaction between VDAC3 and FBXW7. Moreover, FBXW7 expression decreased in Jurkat and CCRF-CEM cells after Rapa treatment, whereas FBXW7 expression in Reh cells was significantly lower than that in the control group after CQ treatment (*p* < 0.05 for all, Figures 3E,F). These findings revealed the interaction between FBXW7 and VDAC3, and FBXW7 protein expression could be regulated by autophagic activity.

### FBXW7 Participated in the Ubiquitination and Degradation of VDAC3

**FIGURE 4:**
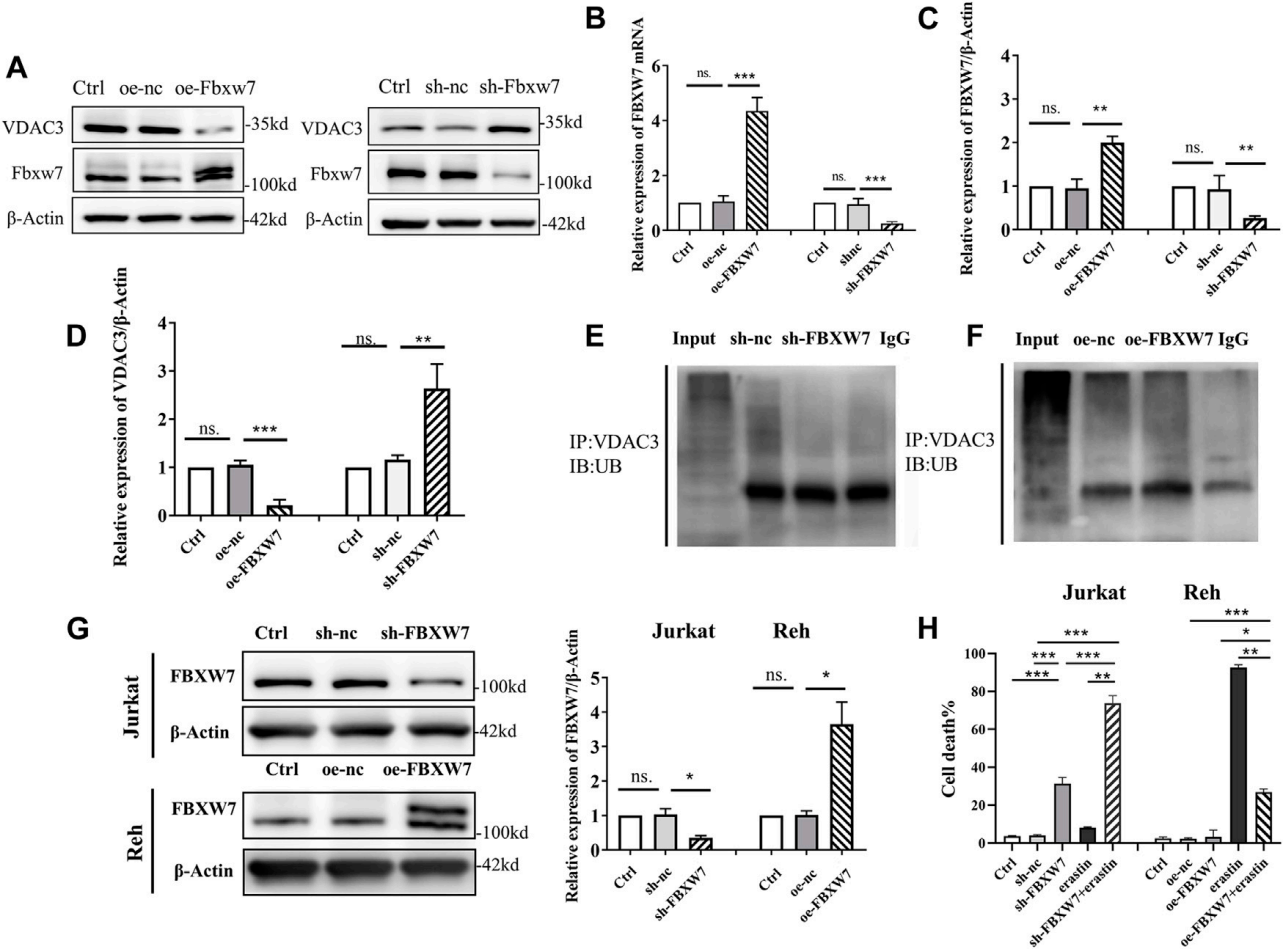
FBXW7 affected the ubiquitination level of VDAC3 and the sensitivity of ALL cells to erastin. **(A)** Confirmation of the transfection efficiency of VDAC3 and FBXW7 proteins in 293T cells with *FBXW7* overexpression/RNAi lentiviral vector by IB. The expression of β-actin was examined as a control. **(B)** Confirmation of transfection efficiency of VDAC3 and *FBXW7* mRNA expression in 293T cells with *FBXW7* overexpression/RNAi lentiviral vector by qRT-PCR. **(C)** Histogram of the FBXW7 protein expression with *FBXW7* overexpression/RNAi lentiviral vector transfection. **(D)** Histogram of the relative expression levels of VDAC3 following *FBXW7* overexpression or knockdown. **(E)** Cells with *FBXW7* silencing were treated with 10 μmol/l MG132 for 6 h and whole cell lysates were subjected to immunoprecipitation with either VDAC3 antibody or normal IgG antibody. **(F)** Cells overexpressing *FBXW7* were treated with 10 μmol/l MG132 for 6 h and whole cell lysates were subjected to immunoprecipitation with either VDAC3 antibody or normal IgG antibody. A ubiquitin antibody was used for IB. **(G)** Confirmation of the transfection efficiency of FBXW7 in ALL cells with *FBXW7* overexpression/RNAi lentiviral vector by IB. Left, expression levels of FBXW7; right, histogram of results. **(H)** Confirmation of the transfection efficiency of VDAC3 and FBXW7 in Reh cells with the *FBXW7* overexpression lentiviral vector by IB. The expression of β-actin was examined as a control. **(H)** Trypan blue staining detection of ALL cell death following different treatments. Left, percentages of trypan blue-positive cells; right, histogram of results. p* <0.05, **p < 0.01, ***p < 0.001, ns. indicates no statistical significance.

To clarify how FBXW7 affects VDAC3 degradation, *FBXW7* was knocked-down or overexpressed in 293T cells. The transfection was effective as confirmed by IB and qRT-PCR (Figures 4A,B). As depicted in Figures 4C,D, VDAC3 protein levels increased significantly after *FBXW7* was silenced (*p* < 0.001) and decreased significantly when *FBXW7* was overexpressed (*p* < 0.001). These results confirmed that VDAC3 protein expression was related to the level of *FBXW7*. Additionally, the levels of VDAC3 ubiquitination were detected by IP and IB assays after *FBXW7* expression was altered. As shown in Figures 4E,F, the ubiquitination of VDAC3 decreased after *FBXW7* silencing and increased after *FBXW7* overexpression, suggesting that FBXW7 degraded VDAC3 via the UPS. Then, *FBXW7* was knocked-down in Jurkat cells and overexpressed in Reh cells. The efficiency of transfection was determined by IB (Figure 4G). As shown in Figure 4H, mortality rates were significantly higher for Jurkat cells treated with sh-*FBXW7* and erastin than in cells treated with sh-*FBXW7* or erastin alone (both *p* < 0.001). Similar results were obtained for Nalm6 and Sup-B15 cells (Supplementary Figure S3). In contrast, oe-*FBXW7* reduced the sensitivity of Reh cells to erastin (*p* < 0.05). These results indicated that the silencing of *FBXW7* increased the sensitivity of ALL cells to erastin.

### Autophagic Activation Enhanced the Therapeutic Effect of Erastin *In Vivo*

**FIGURE 5:**
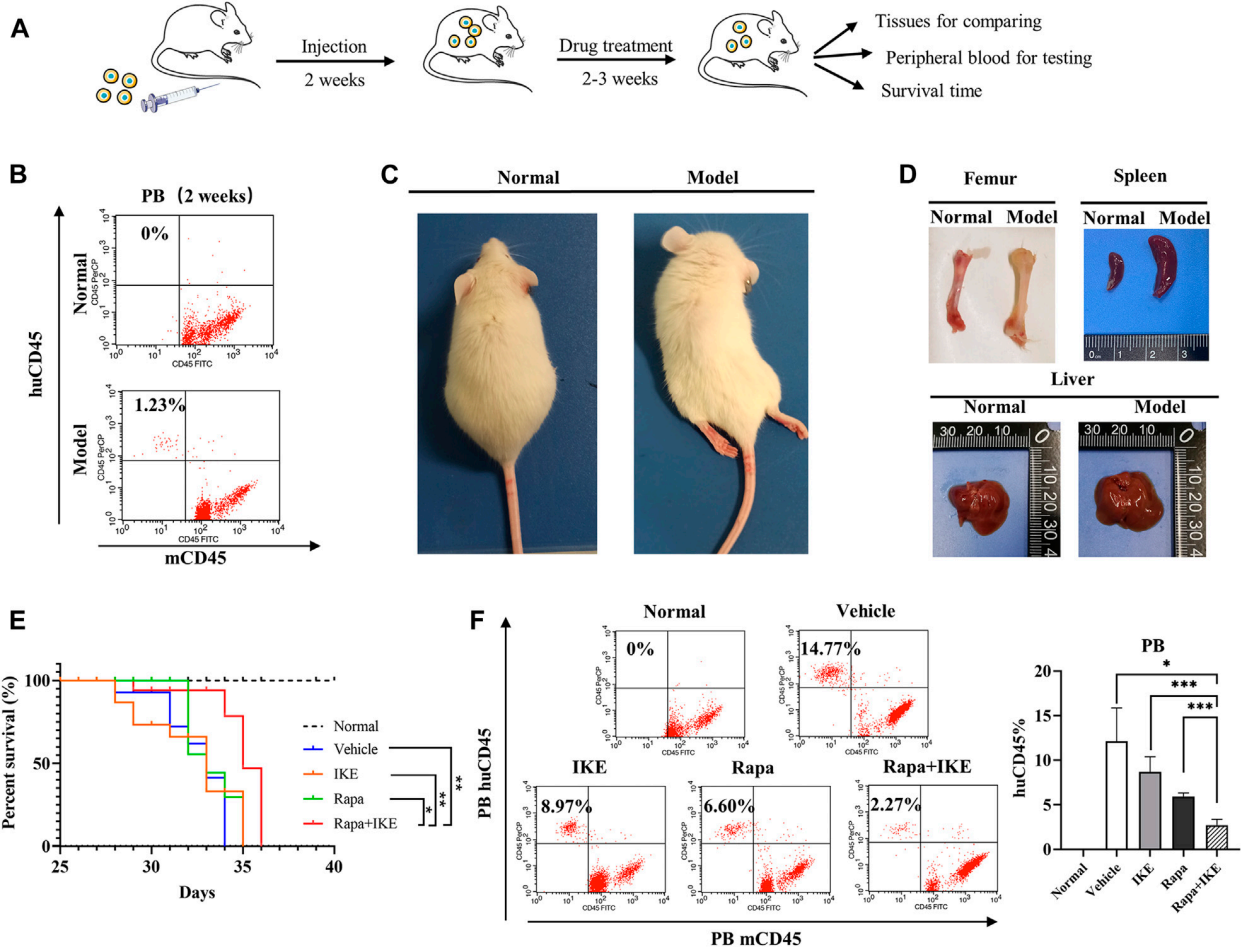
Effect of the combination of rapamycin and erastin *in vivo*. NOD/SCID mice were injected with 5 × 10^6^ CCRF-CEM cells (in 200 µl). Mice were treated with vehicle (2% DMSO + 30% PEG300 + 68% normal saline), Rapa (2 mg/kg), IKE (20 mg/kg), and Rapa (2 mg/kg) + IKE (20 mg/kg) on week 2 after transplantation (*n* = 8/group). **(A)** Schematic overview of *in vivo* drug treatment. **(B)** Flow cytometric detection of human ALL cells (huCD45^+^mCD45^–^) in peripheral blood of mice engrafted with ALL cells (week 2). **(C)** Morphological changes in engrafted mice. **(D)** Representative photographs of livers and spleens from mice engrafted with CCRF-CEM cells after 4 weeks. **(E)** Survival curve of mice with different treatments. **(F)** Flow cytometric detection of human ALL cells (huCD45^+^mCD45^–^) in peripheral blood of engrafted mice following different treatments. Left, percentages of leukemic cells (huCD45^+^mCD45^–^) in the peripheral blood of mice; right, histogram of results. *p < 0.05, **p < 0.01, ***p < 0.001.

To confirm the therapeutic effect of the combination of Rapa with erastin *in vivo*, we used a xenograft mouse model. The erastin-resistant cell line CCRF-CEM was transplanted into NOD/SCID mice (Figure 5A) and huCD45^+^mCD45^–^ cells were evaluated by FCM every week. At week 2, huCD45^+^mCD45^–^ leukemic cells in PB were detected (Figure 5B). After huCD45^+^mCD45^–^ cells reached >1%, Rapa, IKE (erastin for *in vivo* administration), Rapa+IKE, and vehicle were administered once every other day by i.p. injection for 2–3 weeks. After transplantation, animals manifested signs of advanced disease, such as dull hair, arched back, rear limb weakness, or paralysis (Figure 5C). Compared to normal mice, the femur of recipient mice showed a more apparent white appearance and the spleen and liver were significantly enlarged (Figure 5D). These findings indicated that the leukemia engrafted successfully. The frequency of huCD45^+^mCD45^–^ cells in PB of the combination treatment group was significantly lower than that in the Rapa or IKE alone treatment groups (both *p* < 0.05, Figure 5F). Rapa+IKE extended the survival mice with advanced disease compared to the single treatment groups, as shown by Kaplan–Meier curves (Figure 5E). These data suggested that the administration of an autophagy activator could enhance the therapeutic effect of erastin.

## Discussion

ALL is a highly heterogeneous disease with a high risk of failure; in pediatric ALL, up to 25% of patients show relapse after intensive combination chemotherapy. Traditional chemotherapy drugs typically work by inducing apoptosis in cancer cells (Soengas and Lowe 2003; Sarmento-Ribeiro et al., 2012; Zheng 2017). Leukemic cells with the ability to evade apoptosis tend to show chemoresistance, resulting in relapse (Hu and Xuan 2008), and this substantially decreases overall survival in ALL. It is therefore necessary to identify alternative methods to reduce chemoresistance and optimize overall survival. Ferroptosis is a non-apoptotic form of regulated cell death, triggered by ROS generation from accumulated iron and lipid peroxidation. Emerging evidence shows that ferroptosis can be harnessed for cancer therapy. In solid tumors, ferroptosis can eradicate aggressive malignancies resistant to traditional therapies (Ma et al., 2016; Roh et al., 2016; Li et al., 2020). Nevertheless, the application of ferroptosis induction in hematological malignancies is limited. It has been reported that erastin could enhance the sensitivity of acute myeloid leukemia cells to chemotherapy. However, little is known about ALL. Here, we found that ALL cells show different sensitivity to erastin. Various cell lines including B-ALL (Nalm6, Reh), T-ALL (Jurkat, CCRF-CEM, CEM/C1), CML (HL-60), and THP-1cells were evaluated; the majority responded poorly to erastin, except for Reh cells.

Recent evidence suggests that autophagy might facilitate ferroptosis by degrading anti-ferroptosis regulators, revealing the interaction between autophagy and ferroptosis (Liu et al., 2020; Zhou et al., 2020). Notably, our results demonstrated that the combination of Rapa and erastin drastically increased ALL cell death. *In vivo* studies confirmed that the combination of Rapa with erastin significantly prolonged overall survival in a mouse model of ALL. Therefore, it is imperative to determine the mechanism underlying the interaction between autophagy and ferroptosis.

Erastin is the most well-established ferroptosis activator. Ferroptosis induced by erastin is closely related to ROS homeostasis in cells. Yagoda et al. (Yagoda et al., 2007) demonstrated that erastin induced ferroptosis by binding directly to VDAC2/3 to alter the permeability of the outer mitochondrial membrane, which decreased the rate of NADH oxidation and increased ROS production. Although both VDAC2 and VDAC3 are direct targets of erastin, only the functions of VDAC2 have been evaluated in detail (Naghdi and Hajnóczky 2016; Chin et al., 2018). The specific role of VDAC3 remains unclear. Intriguingly, the activation of VDAC3 is closely related to the oxidizing environment and the abundance of ROS in the cytoplasm (Reina et al., 2016). The loss of VDAC3 may affect mitochondrial activity and disrupt ROS signaling pathways, which play a vital role in erastin-induced ferroptosis and are involved in many biological processes (Maldonado et al., 2013; Messina et al., 2014). We showed that autophagy promoted erastin-induced ferroptosis by up-regulating VDAC3 expression in a post-transcriptional manner.

The autophagy lysosome pathway and UPS are the two main pathways for protein quality control in eukaryotes. There is a precise interaction between the two degradation systems. Notably, autophagic activity increases compensatively to relieve the pressure from UPS (Dikic 2017; Pohl and Dikic 2019). Studies have shown that the interaction between the UPS and autophagy can either cause or abolish ferroptosis, depending on the cargo that is degraded (Chen et al., 2021). In this study, the autophagy activator Rapa increased the VDAC3 expression post-transcriptionally in Jurkat and CCRF-CEM cells, while VDAC3 expression was reduced after autophagy blocking in Reh cells. These results revealed that VDAC3 expression was regulated by autophagy and also indicated that the autophagy lysosomal pathway did not play a key role in VDAC3 stability. Additionally, MG132 treatment attenuated the autophagy-induced changes in VDAC3 expression, which demonstrated that VDAC3 degradation was mainly regulated by the UPS, rather than the autophagy lysosomal pathway.

Protein degradation by the UPS requires E3 ligase. Previous research has revealed that the post-transcriptional up-regulation of VDAC3 plays an essential role in improving erastin-induced ferroptosis. Using the UbiBrowser database, FBXW7 was identified as a candidate E3 ligase of VDAC3 in this study; this interaction was confirmed by Co-IP and IB assays. FBXW7 functions in the substrate recognition of E3 ubiquitin ligase and plays vital roles in many physiological processes (Sailo et al., 2019). A close relationship between FBXW7 and autophagy was observed in this study. The protein level of FBXW7 decreased significantly after Rapa administration in Jurkat and CCRF-CEM cells, while it increased after CQ treatment in Reh cells. These findings demonstrated that autophagy also regulated FBXW7 expression in ALL cells. Notably, as an E3 ligase, FBXW7 is engaged in the degradation of many oncoproteins, including NOTCH, MCL1, KLF5, c-JUN, c-MYC, and cyclinE, and participates in many signal transduction pathways (Oberg et al., 2001; Strohmaier et al., 2001; Welcker et al., 2004; Wei et al., 2005; Wertz et al., 2011; Davis et al., 2014; Yumimoto and Nakayama 2020). Recently, FBXW7 has been shown to degrade ZFP36 to regulate sorafenib-induced ferroptosis, revealing that ferroptosis is a therapeutic target for liver fibrosis (Zhang et al., 2020). However, the mechanism by which FBXW7 degrades VDAC3 has not been reported. We predicted that VDAC3 is a substrate of FBXW7 using the UbiBrowser database and verified their interaction by a Co-IP assay in 293T cells (Li et al., 2017). The ubiquitination level of VDAC3 decreased significantly after *FBXW7* silencing, and increased greatly after *FBXW7* overexpression. *FBXW7* mutations were reported in leukemia cell lines. CCRF-CEM cells were reported to the R465C mutation in *FBXW7*, which is the most common mutation in humans, and Jurkat cells G511G, R505C, and Y310Y mutations (Kalender Atak et al., 2012; Squiban et al., 2017). In general, *FBXW7* mutations occur in the WD domain, which affectes substrate stability. The expression of VDAC3 in ALL cell lines and 293T cells was identified. Among these cell lines, VDAC3 expression did not corelate with *FBXW7* mutations. As no specific alterations of VDAC3 expression was observed in CCRF-CEM and Jurkat cells *FBXW7* mutations (Supplementary Figure S4), that VDAC3 stability was less affected by these *FBXW7* mutations, unlike NOTCH or c-MYC. The manipulation of *FBXW7* expression altered the response of ALL cells to erastin in this study. Similar to the results of autophagy activation, silencing *FBXW7* promoted the ferroptosis induced by erastin, demonstrating the critical role of FBXW7 in ferroptosis in ALL. Although several studies have evaluated the function of FBXW7 in ubiquitin degradation (Yeh et al., 2018; Yumimoto and Nakayama 2020), to knowledge, this is the first study the mechanism by which *FBXW7* participates indegrading VDAC3 in ALL. The results showed that autophagy regulated FBXW7 expression. This explains the erastin-induced ferroptosis in ALL cells after autophagy activation, thus providing a new target for clinical. Moreover, the combination of Rapa and erastin prolonged the overall survival, further supporting its clinical value.

## Conclusion

In summary, we demonstrated the mechanism by which autophagy regulated erastin-induced ferroptosis in ALL. We found that FBXW7 participated in degrading VDAC3 by ubiquitination to promote erastin-induced ferroptosis in ALL, explaining the regulatory link between autophagy and ferroptosis. We confirmed the value of the combination of Rapa and erastin for ALL treatment both *in vitro* and *in vivo*.

## Data Availability

The original contributions presented in the study are included in the article/Supplementary Material, further inquiries can be directed to the corresponding authors.
