# Supplementary figures and images for: Autophagy Regulates VDAC3 Ubiquitination by FBXW7 to Promote Erastin-Induced Ferroptosis in Acute Lymphoblastic Leukemia
Source: Front Cell Dev Biol. 2021 Nov 15;9:740884. doi: 10.3389/fcell.2021.740884 (PMC8634639; doi:10.3389/fcell.2021.740884)

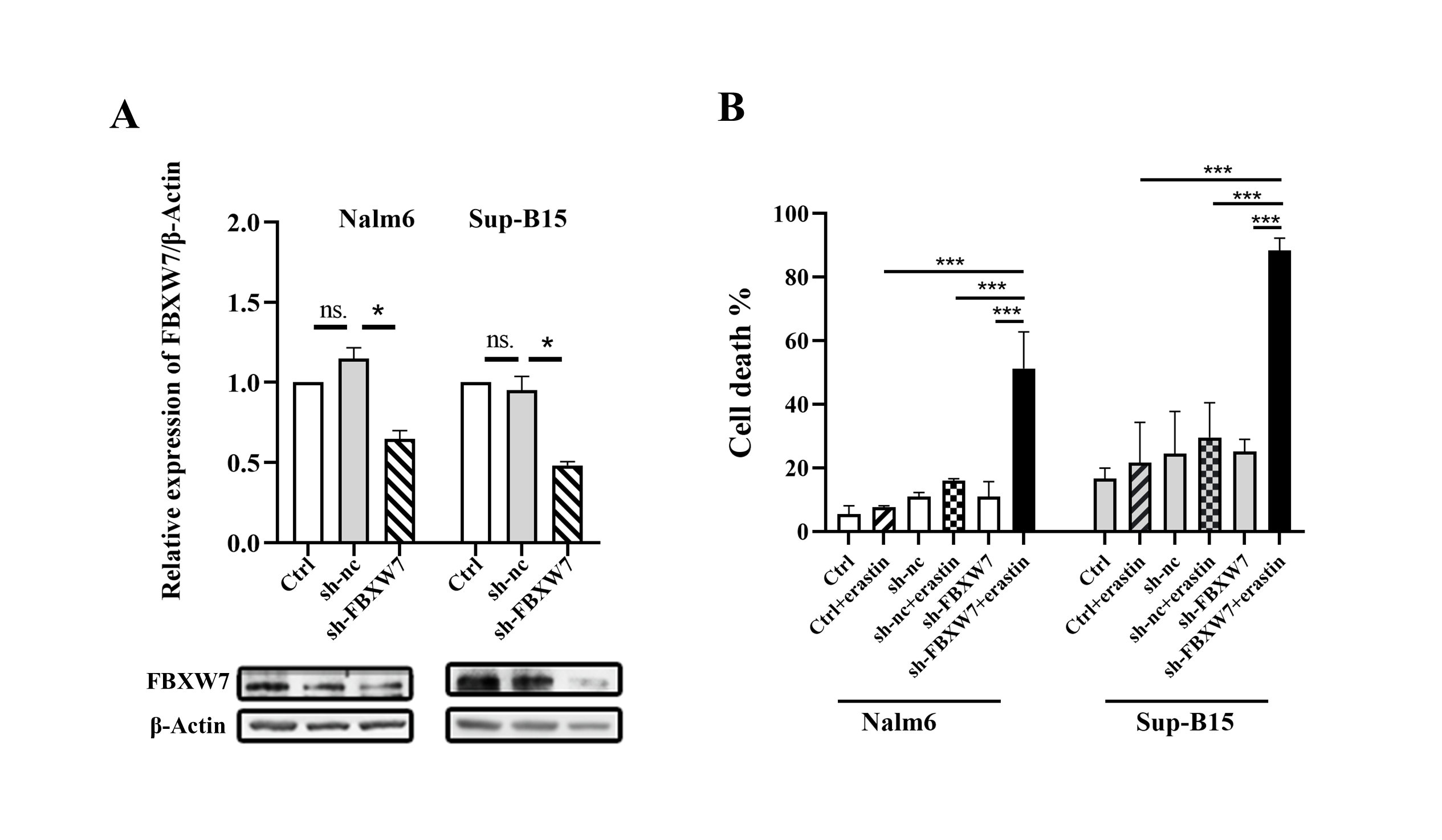

Supplement: Supplementary file 1 [file Image3.TIF]

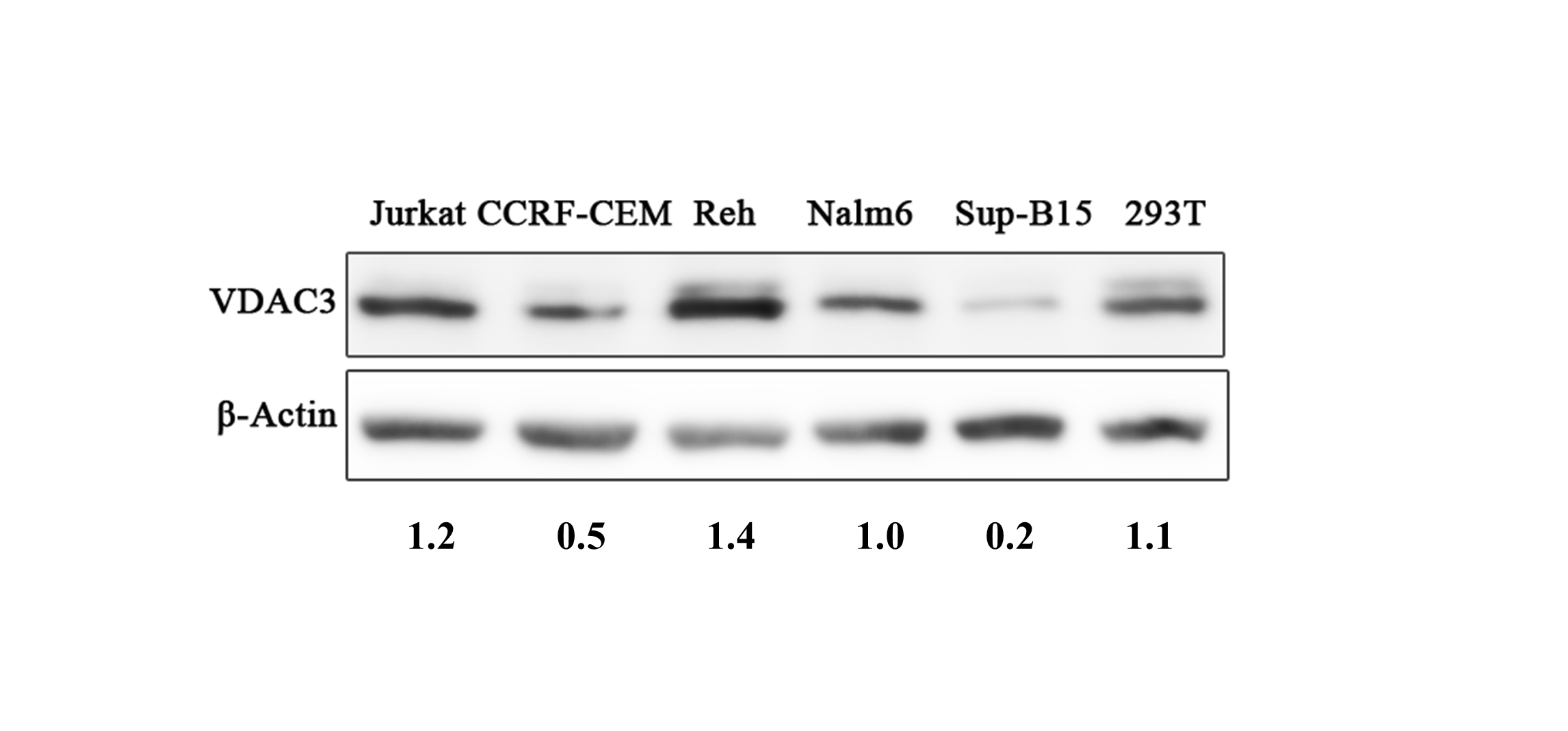

Supplement: Supplementary file 2 [file Image4.TIF]

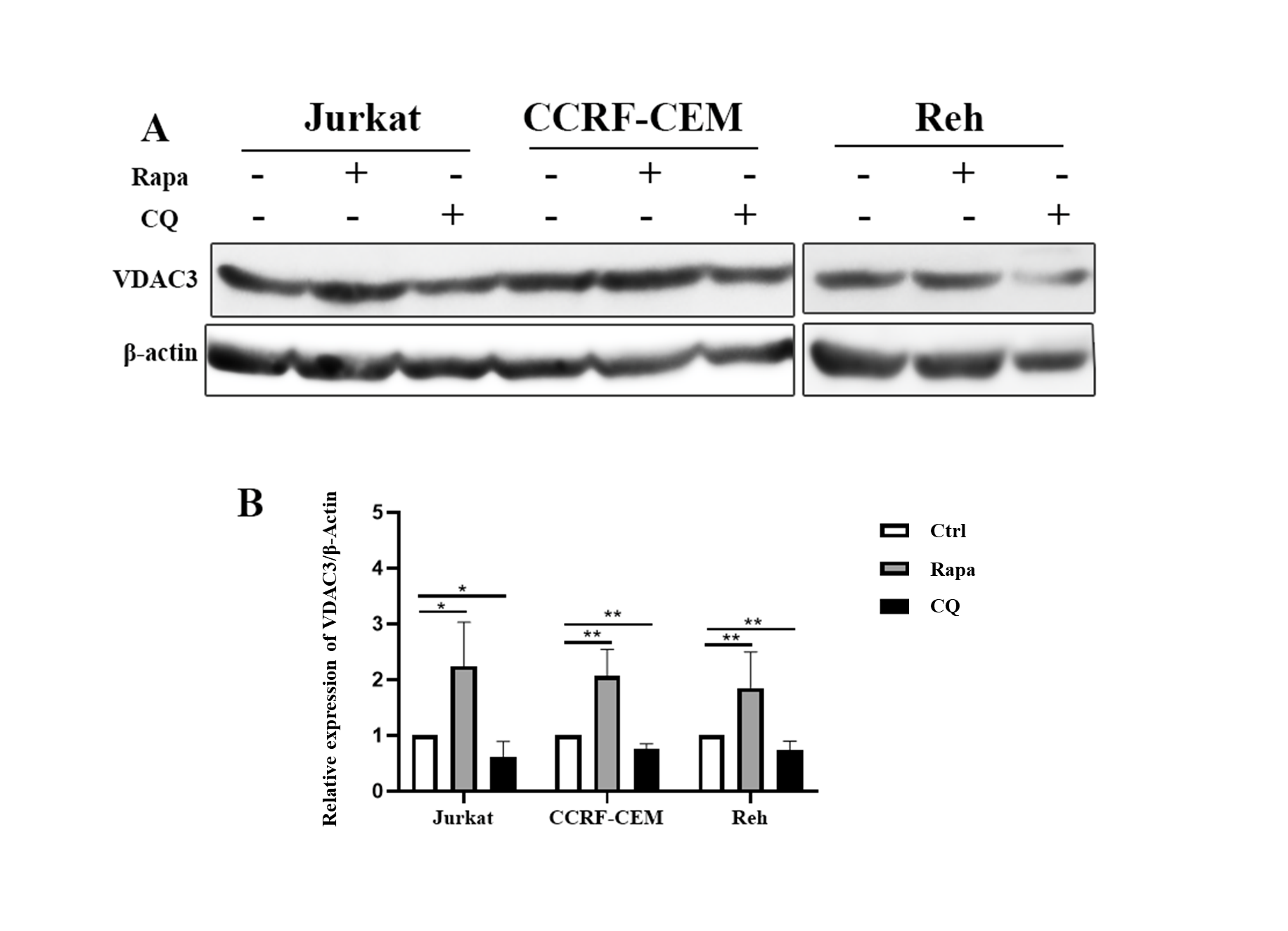

Supplement: Supplementary file 3 [file Image2.TIF]

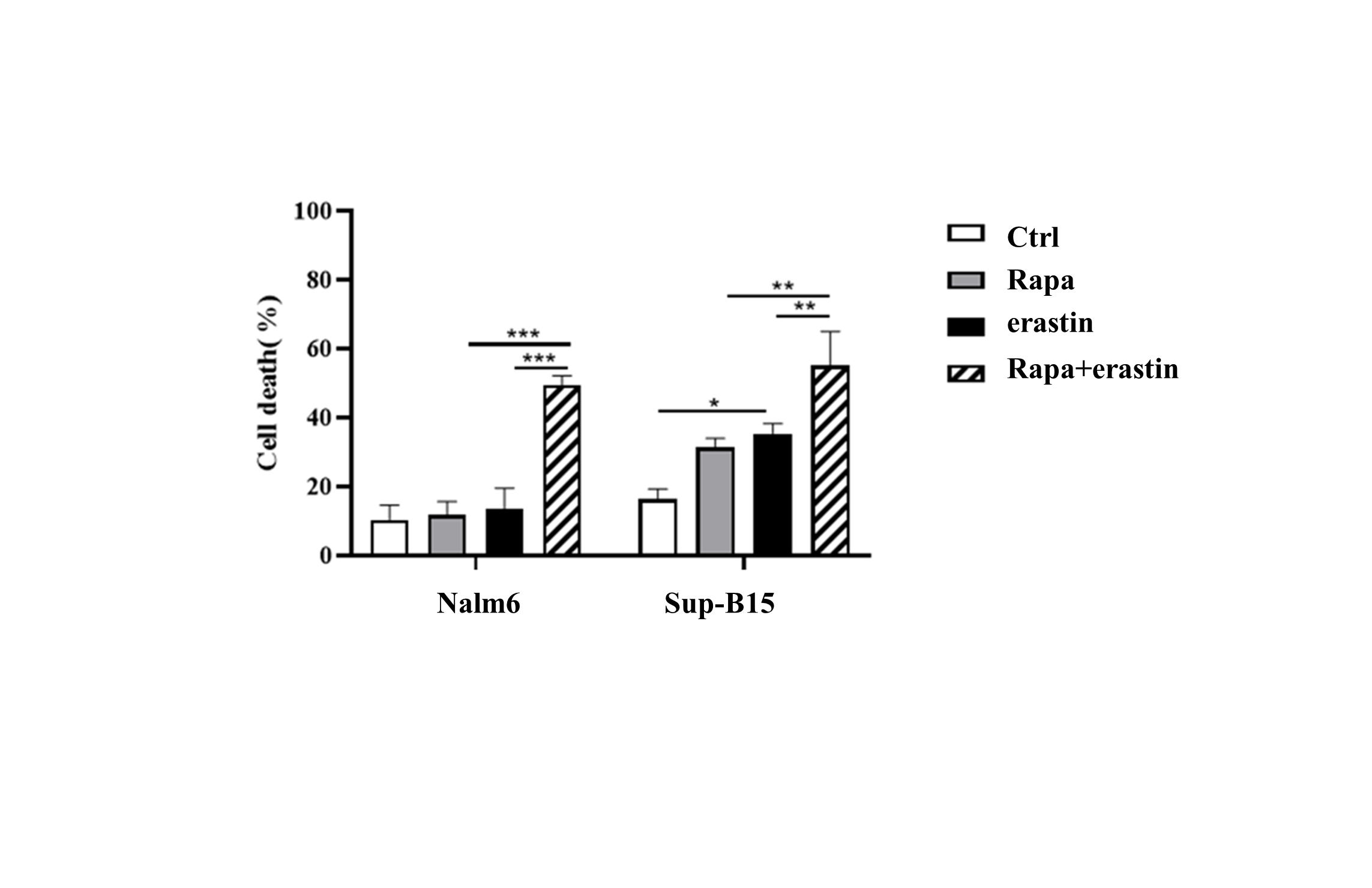

Supplement: Supplementary file 4 [file Image1.TIF]

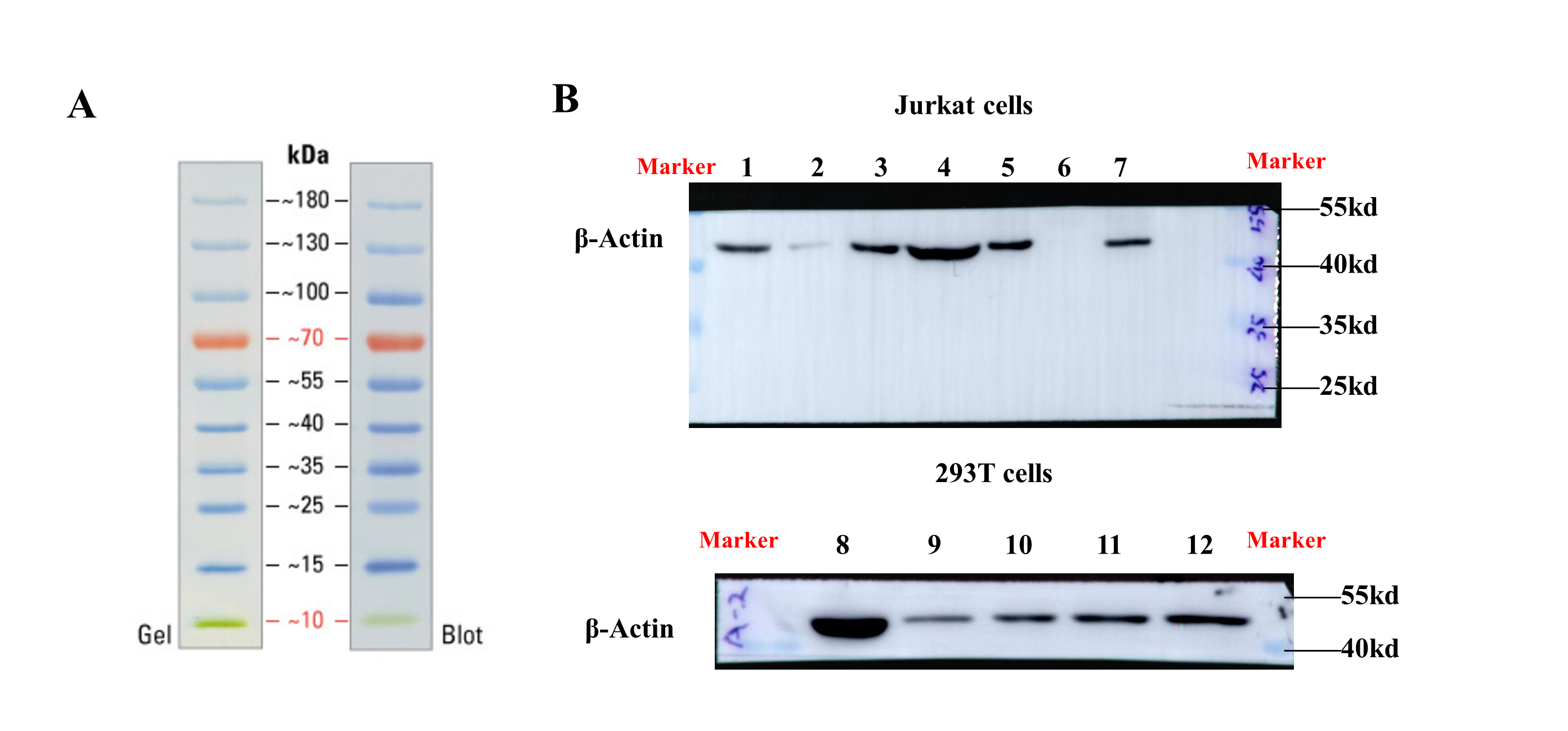

Supplement: Supplementary file 5 [file Image5.TIF]
